# Diagnostic Potential of Serum Circulating miRNAs for Endometriosis in Patients with Chronic Pelvic Pain

**DOI:** 10.3390/jcm14145154

**Published:** 2025-07-21

**Authors:** Tomas Kupec, Julia Wittenborn, Chao-Chung Kuo, Laila Najjari, Rebecca Senger, Philipp Meyer-Wilmes, Elmar Stickeler, Jochen Maurer

**Affiliations:** 1 Department of Gynecology and Obstetrics, University Hospital RWTH Aachen, 52074 Aachen, Germany; juwittenborn@ukaachen.de (J.W.); lnajjari@ukaachen.de (L.N.); rsenger@ukaachen.de (R.S.); phmeyer@ukaachen.de (P.M.-W.); estickeler@ukaachen.de (E.S.); jmaurer@ukaachen.de (J.M.); 2 Genomics Facility, Interdisciplinary Center for Clinical Research (IZKF), University Hospital RWTH Aachen, 52074 Aachen, Germany; chao-chung.kuo@rwth-aachen.de

**Keywords:** endometriosis, lower abdominal pain, serum miRNA, biomarkers, non-invasive diagnosis, early detection, machine learning

## Abstract

**Background**: Endometriosis is a chronic gynecological condition marked by ectopic endometrial-like tissue, leading to inflammation, pain, and infertility. Diagnosis is often delayed by up to 10 years. Identifying non-invasive biomarkers could facilitate earlier detection. MicroRNAs, known for their stability in biological fluids and role in disease processes, have emerged as potential diagnostic tools. This pilot study investigated whether serum miRNA profiling can differentiate endometriosis from other causes of chronic pelvic pain. **Methods**: Serum samples from 52 patients (36 with laparoscopically confirmed endometriosis and 16 controls) treated for chronic pelvic pain at a University Endometriosis Centre were analyzed. High-throughput miRNA sequencing was performed. Feature selection reduced 4285 miRNAs to the 20 most informative MiRNAs. Machine learning models, including logistic regression, decision tree, random forest, and support vector machine, were trained and evaluated. **Results**: Among the tested machine learning models, support vector machine achieved the best overall performance (accuracy 0.71, precision 0.80), while logistic regression and random forest showed the highest AUC values (0.84 and 0.81, respectively), indicating strong diagnostic potential of serum miRNA profiling. **Conclusions**: This study demonstrates the feasibility of using serum miRNA profiling combined with machine learning for the non-invasive classification of endometriosis. The identified miRNA signature shows strong diagnostic potential and could contribute to earlier and more accurate detection of the disease.

## 1. Introduction

Endometriosis is a chronic gynecological disorder characterized by the ectopic presence of endometrial-like tissue outside the uterus, typically on pelvic structures such as the ovaries, fallopian tubes, and peritoneum. This tissue responds to hormonal fluctuations of the menstrual cycle, leading to inflammation, bleeding, adhesions, and scar tissue formation [1]. This condition is associated with symptoms such as lower abdominal pain, dysmenorrhoea, dyspareunia, dysuria, dyschezia, and infertility. Chronic pain often results in reduced productivity, missed educational and work opportunities, and challenges in personal relationships and sexual health.

Endometriosis affects approximately 10% of women of reproductive age [2]. However, many cases remain undiagnosed or unreported, so the prevalence is underestimated.

Fortunately, because of its prevalence and negative impact on quality of life, endometriosis is receiving increasing attention. The main problem with endometriosis is the long time that lies between the onset of symptoms and the correct diagnosis. On average, it takes 10 years for endometriosis to be diagnosed. Surprisingly, this applies to all countries where such investigations have been carried out [3,4,5,6]. The diagnosis is often only made during an infertility investigation, highlighting a significant gap in early detection [3].

Although clinical assessment, gynecological examination, vaginal ultrasound, and detailed medical history are often sufficient for the clinical diagnosis of endometriosis and treatment initiation, inadequate consultation time and limited expertise in managing menstrual pain in routine practice frequently lead to diagnostic delays and inadequate therapy [7].

The chronic nature of endometriosis also raises concerns about overtreatment through repeated surgery, which is often unnecessary [8]. While laparoscopy was once considered the diagnostic gold standard, its use is now limited to selected cases [9]. Despite this, the most common form of endometriosis—peritoneal endometriosis—can only be definitively diagnosed by laparoscopy, highlighting the urgent need for minimally invasive diagnostic methods. Salivary microRNA analysis offers promising noninvasive alternatives, even if further validation is needed before widespread implementation [10].

MicroRNAs (miRNAs), small non-coding RNAs, have been reported as promising biomarkers in the diagnosis of cancer and degenerative diseases [11,12,13]. As miRNAs are present in various body fluids, including serum, urine, and saliva, they offer a good tool for a non-invasive diagnostic approach [10,14,15]. Our preliminary studies demonstrated the stability and high expression levels of miRNAs in serum, suggesting the potential of this body fluid as a diagnostic tool for routine clinical practice [16,17]. According to the findings of previous studies, it seems that focusing on the search for non-invasive diagnostics in the field of miRNA is the right way to promising future results.

The aim of this pilot study is to identify and validate reliable, minimally invasive miRNAs biomarkers for the early and accurate diagnosis of endometriosis. By addressing the limitations of current diagnostic methods, this research seeks to improve the differentiation of endometriosis from other causes of chronic lower abdominal pain. Additionally, it aims to establish miRNA analysis in serum as a practical diagnostic tool for routine clinical use, ultimately reducing delays in diagnosis and improving patient outcomes.

## 2. Materials and Methods

### 2.1. Patient Selection

Data from 52 patients (36 with confirmed endometriosis, 16 patients with excluded endometriosis as controls) who were treated with lower abdominal pain or suspicion of endometriosis at the Endometriosis Centre of RWTH Aachen University Hospital between December 2021 and August 2023 were analyzed.

Prior to the examination, patients completed in the waiting room a standardized medical history form to record demographic data and self-reported symptoms. The diagnosis of endometriosis was made according to the current ESHRE guidelines [9] after a gynecological examination and ultrasound, performed by a senior consultant with extensive experience at a specialized Endometriosis Centre. The examination was followed by a detailed medical consultation, during which the senior consultant and the patient discussed further treatment planning, as well as a counselling and support session on the topic of endometriosis. Concrete endometriosis-specific therapy was planned—endocrine therapy, surgical therapy, analgesia, or fertility treatments.

Patients were eligible for inclusion in the study if they were scheduled for surgical laparoscopy at our Endometriosis Centre during the study period based on a clinical suspicion of endometriosis because of lower abdominal pain symptoms.

Patients with adenomyosis uteri, uterine fibroids, inflammatory bowel disease, or other chronic inflammatory or gynecological conditions were excluded based on clinical evaluation and detailed medical history.

The operation was performed by one of the three surgeons with many years of experience in the Endometriosis Centre at our clinic. During laparoscopic surgery, the whole abdomen was inspected, and in the case of endometriosis, the lesion was removed and histologically confirmed by biopsy. All patients in the endometriosis group had histologically confirmed endometriosis. The control group includes patients who underwent surgery because of suspected endometriosis, but endometriosis was excluded during the surgery.

The severity and localization of endometriotic lesions were classified intraoperatively using the revised #ENZIAN classification system, which provides a standardized framework for describing the extent and distribution of endometriosis [18].

Written informed consent was obtained from all patients involved in the study. The study was conducted in accordance with the Declaration of Helsinki and approved by the Independent Ethics Committee of the Faculty of Medicine at RWTH (ethics vote 206/09) for studies involving humans.

### 2.2. Sampling

Before the operation, a serum sample was taken for miRNA analysis as part of the preoperative routine laboratory examination. A total of fifty-two serum tubes (Greiner Bio-One, Kremsmünster, Austria), each containing 10 mL of whole blood, were collected from all patients. The samples obtained were stored at 4 °C in the Endometriosis Centre until fully clotted. The samples were then centrifuged at 2500× *g* for 10 min. The serum supernatant was carefully pipetted and separated into aliquots, then stored at −80 °C on the same day after registration of the data in the biobank of RWTH University hospital Aachen. The samples were then labelled with a pseudonym until further processing.

### 2.3. Serum Sample Preparation

For miRNA extraction, at least 7 mL of cell-free supernatant was available, of which 4 mL was used for miRNA extraction, and the remaining volume was used as a backup. A total of 52 patients were included (*n* = 36 endometriosis patients and *n* = 16 controls) for final analysis. The miRNA was extracted from each serum sample using the miRNeasy Serum Kit (Qiagen, Hilden, Germany) according to the manufacturer’s instructions.

### 2.4. miRNA Sequencing and Statistical Analysis

Sequencing libraries were prepared with the QIASeq miRNA UDI Library Kit (Qiagen, Hilden, Germany) according to the manufacturer’s instructions. To the recommended 4 µL sample input for biofluids, 1 µL of synthetic miRNAs from the QIASeq miRNA Library QC Kit (Qiagen, Hilden, German) was added as additional quality control. The quality of the libraries was checked on a Bioanalyzer or Tapestation (both Agilent, Waldbronn, Germany), and the libraries were quantified by a Quantus fluorometer (Promega, Madison, WI, USA). All the samples were sequenced on an Illumina NextSeq 500 instrument (Illumina, San Diego, CA, USA) in 72 bp single-end mode. Sequencing yielded a mean coverage of approximately five million reads per sample.

FASTQ files were generated using bcl2fastq v2.3 (Illumina). To facilitate reproducible analysis, samples were processed using the publicly available nf-core/smRNAseq pipeline version 2.3.0 [19] implemented in Nextflow 23.10.1 [20] using Docker 24.0.2 with minimal command. All analyses were performed using custom scripts in R version 4.3.3 using the DESeq2 v.1.38.3 framework [21].

All samples from 36 endometriosis patients and 16 controls were used in the downstream analyses. After normalizing the read counts using DESeq2, we applied cross-validation with five data folds for robust evaluation. Our approach comprises two strategies: first, the algorithm of models (logistic regression, decision three, random forest, SVM (Support Vector Machine)) were used on the more than 4000 miRNAs to capture broad interactions, and second, each model-based feature selection was employed to narrow down relevant miRNAs. We utilized Python tools (version 3.12) such as scikit-learn, numpy, pandas, matplotlib, and seaborn for analysis and visualization, ensuring a comprehensive exploration of miRNA–target dynamics.

GraphPad Prism 10 software was used for statistical evaluation. Student’s *t* test was used to determine significant differences.

No generative AI or AI-assisted technologies were used in the writing or preparation of this manuscript. All content has been generated solely by the authors, who take full responsibility for its accuracy and integrity.

## 3. Results

The mean age of patients with endometriosis was 28.1 years (SD = 7.5; 95% CI: 25.5–30.7), compared to 26.6 years (SD = 6.1; 95% CI: 23.5–29.6) in the control group (*p* = 0.430). Most patients (80.56%) were diagnosed with peritoneal endometriosis (#ENZIAN P), followed by ovarian (#ENZIAN O, 16.67%) and tubal (#ENZIAN T, 5.56%) endometriosis. Deep infiltrating endometriosis was identified in 47.22% of patients, distributed across #ENZIAN A (13.89%), #ENZIAN B (33.33%), #ENZIAN FB (5.56%), and #ENZIAN FI (2.78%). Adenomyosis uteri (#ENZIAN FA) was present in 47.22% of the patients. The patient characteristics are in Table 1.

### 3.1. Analysis of 52 Serum Samples from Women with and Without Endometriosis (With Lower Abdominal Pain)

We implemented miRNA sequencing as an unbiased method to evaluate the currently known miRNA genome from human serum samples. The first objective was to implement reliable and consistent detection of miRNAs in serum. Since this study was designed to evaluate the feasibility of miRNA sequencing from a reasonably small sample volume that can be obtained during a regular outpatient visit, we tested a sample size of 4 mL of serum in this sequencing approach.

### 3.2. Preprocessing

The dataset consisted of 4285 miRNAs as features and 52 serum samples categorized into a positive group and a negative group. Preprocessing steps involved handling missing values (none detected), filtering out rows with 100% zero values (reducing the dataset to 3800 miRNAs), and identifying outliers using z-scores. Exploratory analysis involved descriptive statistics and principal component analysis (PCA) to visualize data variability and clustering patterns. Consistent labelling ensured alignment between serum samples and miRNA features for subsequent analysis. These steps enhanced data quality and provided a foundation for identifying diagnostic biomarkers through advanced modelling techniques.

### 3.3. Feature Selection

To enhance model performance and reduce dimensionality, several feature selection techniques were applied to the initial dataset comprising 3800 features. First, features with low variance were removed using a variance threshold, resulting in the exclusion of 1618 non-informative features. Subsequently, univariate feature selection was employed to identify features with strong individual associations with the target variable, reducing the feature set to 1091 dimensions. Further refinement was achieved through mutual information analysis, which captures both linear and non-linear dependencies between features and the target, yielding 545 selected features. Finally, Recursive Feature Elimination (RFE) was applied to identify the most informative subset, resulting in a final set of 20 features for classification modeling.

### 3.4. Selected Features (20 miRNAs)

**Figure 1:**
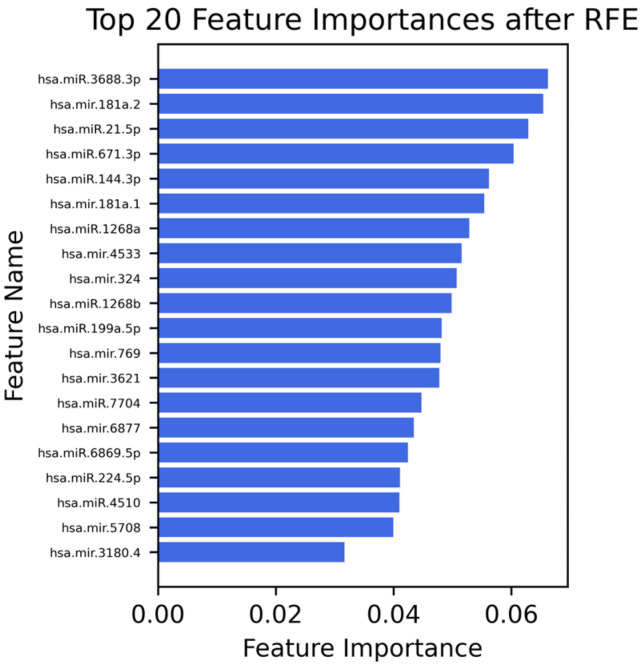
Top 20 most important features identified by Recursive Feature Elimination (RFE) ranked by their contribution to the classification model. These miRNAs demonstrated the highest importance, highlighting their potential relevance as discriminative biomarkers in the studied classification task.

Following the final step of RFE, the top 20 most relevant miRNAs (Figure 1), hsa-miR-3688-3p, hsa-mir-181a-2, hsa-miR-21-5p, hsa-miR-671-3p, hsa-miR-144-3p, hsa-miR-181a-1, hsa-miR-1268a, hsa-mir-4533, hsa-mir-324, hsa-miR-1268b, hsa-miR-199a-5p, hsa-mir-769, hsa-mir-3621, hsa-miR-7704, hsa-mir-6877, hsa-miR-6869-5p, hsa-miR-224-5p, hsa-miR-4510, hsa-mir-5708, and hsa-mir-3180-4, were identified for each classification task. These features represent the most informative biomarkers for distinguishing between endometriosis and the respective control groups across serum samples.

### 3.5. Model Selection

The model selection process involved splitting the dataset into training and test sets while preserving the original class distribution. The dataset consisted of 52 samples—36 from endometriosis patients and 16 from controls. To address class imbalance, we applied stratified splitting using the train_test_split function from scikit-learn, resulting in a training set with 21 positive and 10 negative samples (*N* = 31), and a test set with 14 positive and 7 negative samples (*N* = 21). Each sample included 20 features. K-Fold Cross-Validation was employed during training to enhance model reliability, with folds generated once and applied consistently across all models. Each model was implemented using separate, modular code files, and the same validation folds were reused across all models to ensure consistency and reproducibility during evaluation. Importantly, performance metrics such as accuracy, precision, recall, F1 score, and AUC were calculated exclusively on the independent test set to avoid overfitting and inflated results. This methodological approach provided a reproducible and reliable framework for identifying the most effective miRNA-based classification model.

### 3.6. Model Evaluation

**Figure 2:**
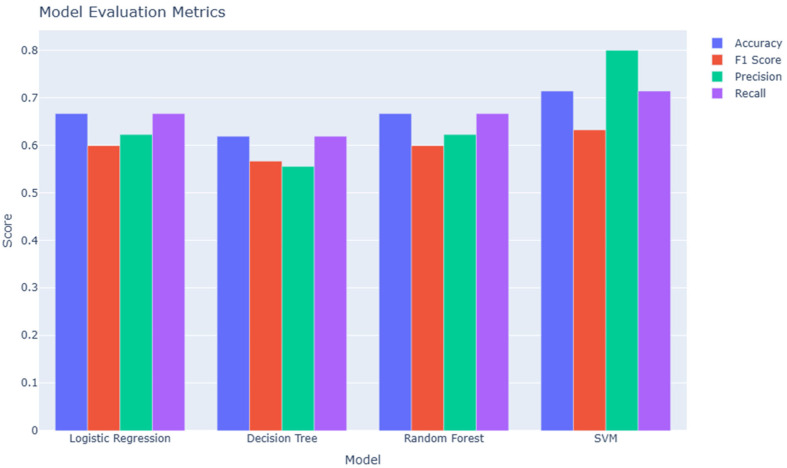
Performance comparison of four machine learning models for endometriosis classification. The models include logistic regression, decision tree, random forest, and support vector machine (SVM). Evaluation metrics include accuracy, F1 score, precision, and recall. The SVM model demonstrated the highest precision and accuracy, while logistic regression and random forest showed balanced performance across all metrics.

Among all models (Figure 2), SVM achieved the highest overall performance, with superior precision (0.80), accuracy (0.71), and recall (0.71), indicating its strong discriminative capability and robustness. Random forest and logistic regression also demonstrated consistent performance across all metrics, slightly outperforming decision tree. While decision tree showed the lowest precision and F1 score, all models maintained recall values above 0.60, suggesting a generally reliable identification of positive cases. These results highlight SVM as the most promising model for the given classification task (Table 2).

**Figure 3:**
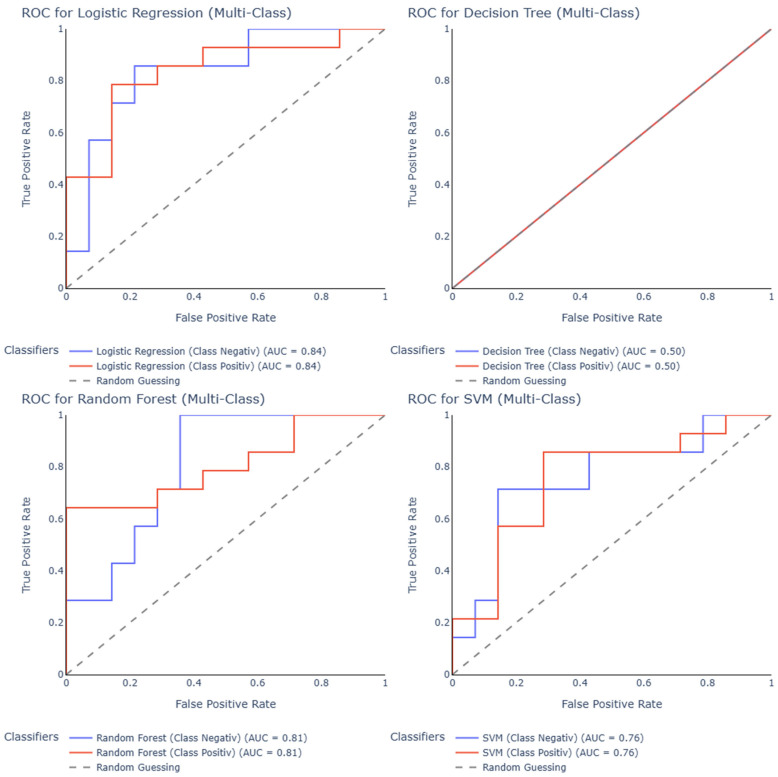
Receiver operating characteristic (ROC) curves for multi-class classification of endometriosis using four different machine learning models: SVM, random forest, decision tree, and logistic regression. Each plot shows the ROC curves for the classification of positive (red) and negative (blue) cases, along with the Area Under the Curve (AUC) values. Logistic regression achieved the highest performance (AUC = 0.84), followed by random forest (AUC = 0.81) and SVM (AUC = 0.76), while the decision tree classifier performed at chance level (AUC = 0.50). The diagonal line represents the performance of a random classifier.

To further assess the discriminatory ability of the models, receiver operating characteristic (ROC) curves and area under the curve (AUC) values were computed for each classifier (Figure 3). The decision tree model exhibited poor discriminative performance, with an AUC of 0.50 for both positive and negative classes, which is equivalent to random guessing. In contrast, logistic regression achieved the highest AUC of 0.84 for both classes, indicating excellent separability between the positive and negative groups. Random forest also performed well, with an AUC of 0.81, closely matching the performance of logistic regression. The SVM classifier yielded a moderate AUC of 0.76, demonstrating good but slightly lower discriminative power. These results confirm the superiority of logistic regression and random forest in distinguishing between the classes based on ROC analysis, while highlighting the limited effectiveness of the decision tree in this task.

## 4. Discussion

Endometriosis remains a diagnostic challenge due to the variability of clinical presentation and the long delay between symptom onset and definitive diagnosis, often averaging ten years. This delay highlights the need for more effective, non-invasive diagnostic tools. Laparoscopy is still the most common way to diagnose and confirm endometriosis, especially in its peritoneal form. The search for non-invasive biomarkers, such as miRNAs, has therefore attracted considerable interest.

In recent years, miRNAs have been identified as potential biomarkers due to their stability in biological fluids and their involvement in various pathophysiological processes. The study by Bendifallah et al. [10] represents a promising step toward non-invasive diagnosis of endometriosis and contributed to the development of a saliva-based test. However, the methodology has been subject to criticism by the gynecological community, primarily due to the lack of independent validation studies and the diagnostic approach—endometriosis was identified by MRI in 46% of cases, including the peritoneal form. As a result, the test is currently not recommended for routine clinical use by gynecological societies.

The search for biomarkers in the serum of endometriosis patients has so far been frustrating [22]. Previous studies have investigated the relevance of miRNA analysis for the diagnosis of endometriosis, but the results are inconsistent because of methodological differences and heterogeneity among control groups [23,24,25,26,27,28].

The aim of our pilot study is to demonstrate the potential of serum miRNA analysis as a promising minimally invasive diagnostic tool for endometriosis. By exploiting the high stability and expression levels of serum miRNAs, this study aimed to lay the groundwork for further research into miRNA-based diagnostics to fill the diagnostic gap and provide a useful alternative to invasive laparoscopy. Using next-generation sequencing in this study holds a significant promise in the search for reliable miRNA-based biomarkers, as it captures both known and previously uncharacterized miRNA variations with high resolution. The results of this study, particularly the high AUC values achieved by the logistic regression and random forest models, highlight the feasibility of using miRNA-based methods for the accurate detection of endometriosis, especially in patients presenting with lower abdominal pain.

The application of advanced statistical and machine learning models to evaluate the diagnostic potential of serum miRNAs yielded promising results. Among the tested models, SVM demonstrated the best overall performance, achieving the highest precision (0.80) and accuracy (0.71), while logistic regression and random forest exhibited the strongest discriminative power, with AUC values of 0.84 and 0.81, respectively. These findings support the feasibility of miRNA-based classification in a clinical context and highlight the complementary strengths of different modeling approaches. While SVM excelled in identifying positive cases, logistic regression and random forest provided robust class separation, making them well-suited for non-invasive endometriosis diagnostics. Given the model interpretability and potential for clinical translation, these results offer a strong foundation for further optimization and validation in larger cohorts.

Using next-generation sequencing, we could identify in serum the 20 most informative MiRNAs for our diagnostic approach. Among the top 20 miRNAs identified in this study, several have established links to endometriosis. Notably, hsa-miR-21-5p is one of the most consistently reported miRNAs in endometriosis research, known for its upregulation in both endometriotic lesions and serum. It plays a key role in promoting cell proliferation, angiogenesis, and inhibiting apoptosis via pathways such as TIMP3–PI3K/Akt/mTOR [29,30,31,32]. Similarly, hsa-miR-224-5p and hsa-miR-199a-5p have been included in diagnostic panels as promising non-invasive biomarkers, with altered expression in patients with endometriosis and involvement in key pathogenic pathways [33,34]. Although direct associations have not yet been established for several other miRNAs in the panel—such as miR-3688-3p, miR-671-3p and miR-144-3p—their reported relevance to regulatory pathways like PI3K/Akt, MAPK, and TGF-β signaling suggests a potential role in disease pathophysiology [10,35,36,37]. Together, these findings support the biological validity of the selected miRNA panel and reinforce the potential of serum miRNA profiling for non-invasive endometriosis diagnosis.

Importantly, a well-performing, non-invasive miRNA-based test could significantly reduce the diagnostic delay, lower the number of unnecessary repeated surgeries and hospital stays, and minimize productivity losses at work or during education. Therefore, despite current limitations, the long-term clinical and socioeconomic benefits highlight the strong potential of integrating miRNA analysis into routine practice as a rapid, reliable, and cost-effective alternative to laparoscopy.

This pilot study has several limitations. Most notably, the relatively small sample size (*n* = 52) limits the generalizability of the findings and reduces statistical power. Although the control group consisted of patients in whom endometriosis was laparoscopically excluded, it may still represent a heterogeneous population with various etiologies of pelvic pain, potentially confounding biomarker specificity. Additionally, the absence of an external validation cohort limits the robustness of the current findings. Future studies involving larger, independent cohorts are essential to validate the identified miRNA signature and confirm its clinical applicability.

Our study highlights several strengths that advance the field of endometriosis diagnostics. By focusing on serum miRNAs as a minimally invasive biomarker, the research addresses the need for alternative diagnostic tools to laparoscopy. The use of robust and standardised methodologies, including miRNA sequencing and advanced data analysis pipelines, ensures consistency and reproducibility of these results. The comprehensive feature selection process further refines the dataset and enhances reliability. In addition, the implementation of various machine learning models, particularly logistic regression, demonstrates a thoughtful approach to identifying effective statistical strategies to reach the best possible model. With clinical relevance at its core, the study design incorporates real-world scenarios by analyzing serum samples from patients with confirmed and excluded endometriosis, providing a solid foundation for translational applications. A major strength of our study is the rigorous selection of a well-defined study cohort, established through strict inclusion criteria that encompassed all patients presenting with chronic pelvic pain and suspected endometriosis. Accurate diagnosis of endometriosis in this population remains one of the greatest challenges in clinical practice. All patients with endometriosis were diagnosed and histologically confirmed through laparoscopy, while endometriosis was also excluded via laparoscopy in the control group. The surgeries were performed by an experienced surgeon at the University Hospital in Germany. Our analysis compared two distinct groups: women with histologically confirmed endometriosis and those with chronic lower abdominal pain in whom endometriosis was excluded by laparoscopy. This differentiation is a significant challenge in routine diagnosis and treatment of chronic abdominal pain. It is essential to distinguish between these two groups to enable early treatment and to avoid unnecessary surgery.

## 5. Conclusions

In conclusion, this pilot study demonstrates that serum miRNA sequencing holds promise as a non-invasive approach for identifying diagnostic biomarkers of endometriosis. The selected miRNA signature successfully distinguished between patients with endometriosis and those with similar symptoms but without histological confirmation. Although further validation in larger, independent cohorts is required, this approach has potential clinical value for earlier diagnosis and improved management of endometriosis.

## Figures and Tables

**Table 1:** Characteristics of patients with endometriosis.

	Mean (SD)
Age (Years)	28.09 (7.46)
Location of Endometriosis Diagnosis (#ENZIAN)	Number of Patients *n*
#ENZIAN P (Peritoneal)	29
#ENZIAN O (Ovary)	6
#ENZIAN T (Tube)	2
Deep infiltrating endometriosis	
#ENZIAN A	5
#ENZIAN B	12
#ENZIAN C	0
#ENZIAN FB	2
#ENZIAN FI	1

**Table 2:** Performance metrics for logistic regression, decision tree, random forest, and support vector machine.

Model	Accuracy	Precision	Recall	F1 Score
Logistic Regression	0.667	0.623	0.667	0.600
Decision Tree	0.619	0.556	0.61	0.567
Random Forest	0.667	0.623	0.667	0.600
SVM	0.714	0.800	0.714	0.632

## Data Availability

The data presented in this study are available on reasonable request from the corresponding author.
